# Addressing Ebola-related Stigma: Lessons Learned from HIV/AIDS

**DOI:** 10.3402/gha.v7.26058

**Published:** 2014-11-07

**Authors:** Mariam Davtyan, Brandon Brown, Morenike Oluwatoyin Folayan

**Affiliations:** 1 Department of Population Health & Disease Prevention, University of California, Irvine, CA, USA; 2 Department of Child Dental Health, Institute of Public Health, Obafemi Awolowo University, Ile-Ife, Nigeria

**Keywords:** HIV, Ebola, stigma, healthcare

## Abstract

**Background:**

HIV/AIDS and Ebola Virus Disease (EVD) are contemporary epidemics associated with significant social stigma in which communities affected suffer from social rejection, violence, and diminished quality of life.

**Objective:**

To compare and contrast stigma related to HIV/AIDS and EVD, and strategically think how lessons learned from HIV stigma can be applied to the current EVD epidemic.

**Methods:**

To identify relevant articles about HIV/AIDS and EVD-related stigma, we conducted an extensive literature review using multiple search engines. PubMed was used to search for relevant peer-reviewed journal articles and Google for online sources. We also consulted the websites of the World Health Organization (WHO), Centers for Disease Control and Prevention (CDC), and the National Institutes of Health to retrieve up-to-date information about EVD and HIV/AIDS.

**Results:**

Many stigmatizing attitudes and behaviors directed towards those with EVD are strikingly similar to those with HIV/AIDS but there are significant differences worthy of discussion. Both diseases are life-threatening and there is no medical cure. Additionally misinformation about affected groups and modes of transmission runs rampant. Unlike in persons with EVD, historically criminalized and marginalized populations carry a disproportionately higher risk for HIV infection. Moreover, mortality due to EVD occurs within a shorter time span as compared to HIV/AIDS.

**Conclusions:**

Stigma disrupts quality of life, whether it is associated with HIV infection or EVD. When addressing EVD, we must think beyond the immediate clinical therapeutic response, to possible HIV implications of serum treatment. There are emerging social concerns of stigma associated with EVD infection and double stigma associated with EVD and HIV infection. Drawing upon lessons learned from HIV, we must work to empower and mobilize prominent members of the community, those who recovered from the disease, and organizations working at the grassroots level to disseminate clear and accurate information about EVD transmission and prevention while promoting stigma reduction in the process. In the long run, education, prevention, and a therapeutic vaccine will be the optimal solutions for reducing the stigma associated with both EVD and HIV.

A number of diseases have had devastating impacts on communities, societies, and civilizations throughout history. The plague, cholera, and leprosy, for instance, have contributed to substantial mortality and morbidity around the world (1, 2). These diseases are also particularly noteworthy for the social stigma they carry and the poor outcomes associated with that stigma (3, 4, 5) . More recent epidemics such as HIV/AIDS and Ebola Virus Disease (EVD) have not only led to significant loss of life but also to stigma which has continued despite global efforts to control both epidemics. Communities affected by HIV/AIDS and EVD-related stigma have suffered isolation and ostracism, physical violence, and diminished quality of life (6). While multiple interventions to reduce HIV/AIDS-related stigma have been developed and implemented, prejudice and discrimination associated with having EVD has received much less attention (7, 8). In this paper, we draw upon lessons learned from HIV/AIDS-related stigma reduction interventions and their possible application to EVD.

## Defining stigma

Early theorists of stigma including Erving Goffman in his 1963 work ‘Notes on the Management of Spoiled Identity’, defined stigma as ‘an attribute that is deeply discrediting’ and one that precludes an individual from full social acceptance (9). Furthermore, the stigmatized person, as a result of this attribute, is reduced from a whole individual to a tainted and discounted one (9). Other definitions of stigma include an attribute or characteristic that is contrary to a norm of a ‘social unit’ (10), conveys a social identity that is devalued (11), and an act of labeling, stereotyping, separation, status loss, and discrimination of someone possessing that attribute or characteristic (12). Stigma related to EVD and HIV/AIDS refers to derogatory attitudes, beliefs, and behaviors directed toward people living with the diseases and those presumed to be infected (8, 13, 14).

## Similarities and Differences between HIV/AIDS- and EVD-related Stigma

Stigma in the context of HIV/AIDS is a prevailing stressor with incapacitating consequences (15). Historically, HIV/AIDS has been attributed to personal irresponsibility and behaviors and lifestyles that are socially unacceptable (16). However, diseases do not exist in a vacuum but are products of broader structural processes such as poverty, racism, and gender inequality (17, 18). These factors individually and collectively shape human vulnerability to disease and its distribution as well as dictate attitudes, beliefs, and behaviors toward certain groups (16). In the context of HIV/AIDS, stigmatized groups have included people who inject drugs, sex workers, and men who have sex with men (14). Stigmatizing attitudes and behaviors, including patient blaming and neglect, refusal/denial of care, and irrational and inappropriate fear of contagion directed toward impacted populations have not only devastated familial, social, and economic relationships and infrastructures but they have also interfered and created colossal barriers to access, prevention, and treatment (17, 19–23) . More specifically, HIV/AIDS-related stigma has led to avoidance of healthcare, reduced adherence to antiretroviral medications, increased HIV symptomatology, and emergence of mental health pathologies including depression (15, 24, 25).

Stigma in the context of EVD is equally disconcerting as it also originates from structural inadequacies, including poverty, lack of education, and political conflict (26). These factors combined with cultural practices subsequently influence attitudes, beliefs, and behaviors with respect to disease transmission (27). During the 2000 and 2001 EVD epidemics in Uganda for example, harassment, rejection, and abandonment of individuals with EVD were common occurrences (8). Those in contact with EVD patients were also victimized with some being prevented from returning to their homes and communities, and their properties destroyed (28). Children were also not spared. There are reports of children orphaned by EVD who remain sero-negative but have not been taken up for care by families and communities out of fear of contagion (29).

As of October 15, 2014, the Centers for Disease Control and Prevention (CDC) reported 8,973 cases of EVD in West Africa and 4,484 deaths (30). The current epidemic affects Liberia, Guinea, and Sierra Leone and has been identified as the worst epidemic in the history of the disease, with a survival rate of 53% (31, 32). Those who have survived the current epidemic face the stigma of having had EVD and this is consistent with previous research findings (8, 28, 33). According to the International Federation of Red Cross and Red Crescent Societies, individuals cured of EVD with certification of clearance by medical authorities have been prohibited from returning to their homes, and shunned by their communities, friends, family, and co-workers (34). Some survivors have also reported threats of violence from their community (35, 36). Instances of civil unrest and violence against healthcare workers and educators have also been reported in addition to killings (37, 38).

Stigmatizing attitudes and behaviors directed towards those with EVD are similar to HIV/AIDS in many important ways. For instance, the idea that EVD only affects certain groups such as poor Africans and African immigrants is comparable to HIV/AIDS as it was attributed to homosexuality in the early days of the epidemic (39, 40). Additionally, EVD and HIV/AIDS have both been categorized as divine retribution for committing undesirable acts (28, 41). Other similarities include irrational and unfounded fear of contracting the pathogen through mechanisms that have not been scientifically indicated, stigmatization of people who associate with those living with the diseases in question (13, 33, 42, 43) and the perception that both diseases are the result of propaganda for population control (44, 45).

Despite the aforementioned similarities, there are also significant differences between EVD- and HIV/AIDS-related stigma. Unlike EVD, persons who are already criminalized and stigmatized by society (men who have sex with men, male and female sex workers, and people who inject drugs) are at disproportionately higher risk for HIV infection (46). Also, while some patients are able to survive EVD and clear the virus after some time, the stigma associated with having had the disease is not alleviated (8, 47, 48). In addition, EVD mortality occurs within a short time span compared to HIV/AIDS and therefore public concern about EVD infection remains elevated (49).

## Addressing Ebola-related Stigma: Lessons Learned from HIV/AIDS

Stigma disrupts the quality of life of affected persons, whether it is associated with HIV infection or EVD. To address stigma in the context of EVD, we can draw upon lessons learned from HIV, consider and perhaps apply strategies that have shown promise. To disseminate accurate information to communities impacted by EVD in ways that facilitate translation of information into action, recruitment and training of Popular Opinion Leaders (POL), may be beneficial (50). POL include members of a community who are highly respected and who may have the ability to mobilize people to work for a common goal, in this case to fight stigma (51). Multi-level community interventions that include accurate information about disease transmission, skill-building, counseling and support, and testimonials from persons who survived the disease as well as those who took care of EVD patients may also be effective (52). Collective strategies such as social activism, capacity, resilience and skill building, and empathy-based contact promoting programs have been beneficial in the fight against HIV/AIDS-related stigma and can be tailored for addressing EVD-related stigma (13, 53, 54).

On a more broader scale, we must work diligently to empower community and religious leaders, persons affected by the disease, and organizations working at the grassroots level to disseminate clear and concise information about EVD transmission and prevention, while also promoting stigma reduction (50). In developing countries, including those affected by the current EVD epidemic, community-based stigma reduction initiatives have been far more effective in reducing HIV/AIDS-related stigma than individual-level programs (55). Building on these findings, we can construct, enhance, and evaluate similar community-based programs for the reduction of stigma associated with EVD (56). For those affected by EVD, it would equally be important to address their capacity to deal with the social stigma they have to face. Using strategies like counseling, training on coping skills, and promoting public contact with persons who have had EVD in an effort at inducing empathy may help reduce EVD-related stigma.

In the long run, education, prevention, and a therapeutic vaccine are the optimal solutions for reducing the stigma associated with both EVD and HIV. However, utilizing the aforementioned strategies as part of community-based advocacy programs may help reduce EVD-related stigma. This approach may be particularly feasible because major donors and stakeholders such as the Gates Foundation, CDC, NIH, and WHO are involved in HIV prevention programs in affected countries are also currently assisting with the EVD response.
